# Quantitative Analysis of Fungal Contamination of Different Herbal Medicines in China

**DOI:** 10.3390/toxins16050229

**Published:** 2024-05-15

**Authors:** Gang Wang, Mingyue Jiao, Junqiang Hu, Yiren Xun, Longyun Chen, Jianbo Qiu, Fang Ji, Yin-Won Lee, Jianrong Shi, Jianhong Xu

**Affiliations:** 1 School of Food and Biological Engineering, Jiangsu University, Zhenjiang 212013, China; wanggang2015@jaas.ac.cn (G.W.); jiaomy2021@163.com (M.J.); xunyr2017@163.com (Y.X.); clyun99@163.com (L.C.); 2 Jiangsu Key Laboratory for Food Quality and Safety-State Key Laboratory Cultivation Base, Ministry of Science and Technology/Key Laboratory for Agro-product Safety Risk Evaluation (Nanjing), Ministry of Agriculture and Rural Affairs/Key Laboratory for Control Technology and Standard for Agro-Product Safety and Quality, Ministry of Agriculture and Rural Affairs/Collaborative Innovation Center for Modern Grain Circulation and Safety/Institute of Food Safety and Nutrition, Jiangsu Academy of Agricultural Sciences, Nanjing 210014, China; 2021216027@stu.njau.edu.cn (J.H.); 20120027@jaas.ac.cn (J.Q.); jifang625@126.com (F.J.); jianrong63@126.com (J.S.); 3 Key Laboratory of Agricultural Environmental Microbiology, Ministry of Agriculture, College of Life Sciences, Nanjing Agricultural University, Nanjing 210095, China; 4 Department of Agricultural Biotechnology, Seoul National University, Seoul 08826, Republic of Korea; lee2443@snu.ac.kr

**Keywords:** mycotoxin, phytopathogenic fungi, herbal medicine, ITS sequencing, RT-qPCR, LC-MS/MS

## Abstract

Herbal medicines are widely used for clinical purposes worldwide. These herbs are susceptible to phytopathogenic fungal invasion during the culturing, harvesting, storage, and processing stages. The threat of fungal and mycotoxin contamination requires the evaluation of the health risks associated with these herbal medicines. In this study, we collected 138 samples of 23 commonly used herbs from 20 regions in China, from which we isolated a total of 200 phytopathogenic fungi. Through morphological observation and ITS sequencing, 173 fungal isolates were identified and classified into 24 genera, of which the predominant genera were *Fusarium* (27.74%) and *Alternaria* (20.81%), followed by *Epicoccum* (11.56%), *Nigrospora* (7.51%), and *Trichocladium* (6.84%). Quantitative analysis of the abundance of both *Fusarium* and *Alternaria* in herbal medicines via RT-qPCR revealed that the most abundant fungi were found on the herb *Taraxacum mongolicum*, reaching 300,000 copies/μL for *Fusarium* and 700 copies/μL for *Alternaria*. The in vitro mycotoxin productivities of the isolated *Fusarium* and *Alternaria* strains were evaluated by using liquid chromatography–tandem mass spectrometry (LC-MS/MS), and it was found that the *Fusarium* species mainly produced the acetyl forms of deoxynivalenol, while *Alternaria* species mainly produced altertoxins. These findings revealed widely distributed fungal contamination in herbal medicines and thus raise concerns for the sake of the quality and safety of herbal medicines.

## 1. Introduction

Herbal medicines are widely used for the prevention and treatment of various diseases, such as psychological [1], gynecological [2], gastrointestinal [3], cardiovascular [4], and respiratory diseases [5]. Herbal medicines are highly effective at regulating and repairing the immune system during COVID-19 infection [6,7]. Medicinal herbs have been used as dietary supplements for centuries, and more than 80% of the world’s population uses herbs as a primary medical resource [8].

As herbs are in direct contact with the soil during cultivation, they are susceptible to fungal colonization, and during harvesting, preparing, storing, and transporting, herbs can also be contaminated by a variety of pathogenic fungi, leading to mold and the production of mycotoxins. Mycotoxins are toxic secondary metabolites produced by fungi such as *Aspergillus*, *Penicillium*, and *Fusarium* [9] that have a variety of toxic effects, including teratogenicity, carcinogenicity, immunotoxicity, and neurotoxicity [10]. At present, more than 400 mycotoxins have been characterized and identified, among which the most dangerous to human and animal health are aflatoxins, trichothecenes, zearalenone, fumonisins, and ochratoxins [11]. The presence of toxins produced by fungal contamination seriously affects the quality and safety of herbal medicines [12]. The occurrence of mycotoxins as well as pesticide and heavy metal residues in herbal medicines poses a serious threat to human health. The occurrence of mycotoxins in medicinal herbs has been reported in many cases.

In a comprehensive review, forty-seven toxic compounds, including mycotoxins, pesticides and heavy metal residues, were identified from 55 species of 46 plant families that exhibit hepatotoxicity, cardiovascular toxicity, and central nervous system and digestive system toxicity, which seriously jeopardize the health of all human beings, and this phenomenon has triggered a global focus on the quality and safety of herbal medicines [13]. A total of 240 samples of 80 Chinese herbal medicines were analyzed for mycotoxin contamination, of which 62.92%, 36.25%, and 64.17% of the samples were contaminated by AFs, OTs, and FBs, respectively, and 24.17% of the herbal medicines had toxin contents exceeding the maximum limits in the European Pharmacopoeia [14]. A total of 30% and 26.7% of the 10 common herbs in Pakistan were contaminated with AFs and OTAs, respectively, with 70% of the samples containing toxin levels exceeding the permissible limits [15]. Of the 34 herbs commonly available in the South African market, 10 were contaminated with mycotoxins to varying degrees, with AFBs, FBs, OTA, and ZEN detected [16]. A total of 42% of the 140 herbal teas sold in Latvia were contaminated with 1–16 mycotoxins, of which AFBs were detected in 32% of the samples, and high contents of DON were detected in 63% of the samples [17]. A total of 126 fungal strains were isolated from 15 common medicinal materials collected in China. *Aspergillus* and *Penicillium* were the main components, and all of them produced mycotoxins to varying degrees [18]. According to an evaluation of fungal contamination in 91 Brazilian medicinal plants, approximately 89.9% of the isolated strains belonged to the genera *Aspergillus* and *Penicillium*, and 21.97% of them had the ability to produce AFBs [19].

Currently, more studies have been conducted on the risk monitoring of mycotoxins in herbal medicines, but less research has been conducted on the diversity and composition of fungal communities on the surfaces of herbal medicines; therefore, the isolation and identification of contaminating fungi in herbal medicines are highly important for identifying the sources of potential mycotoxins in herbal medicines.

The aim of this study is to assess the fungal contamination on different herbal medicines in China and evaluate the mycotoxin productivity of the isolated fungi. Herein, the contaminated fungi were isolated, purified, and cultured from herbal medicines collected from different areas of China, and the fungal strains were identified by traditional morphological and molecular biological methods. Moreover, a real-time quantitative PCR method was established to quantitatively detect the dominant mycoflora, providing a theoretical basis for the early warning of the risk of fungal contamination by herbal medicines. Additionally, the mycotoxin production capacity of the isolated *Fusarium* and *Alternaria* strains was determined by LC-MS/MS.

## 2. Results

### 2.1. Diversity and Composition of Fungal Communities in Herbal Medicines

**Figure 1:**
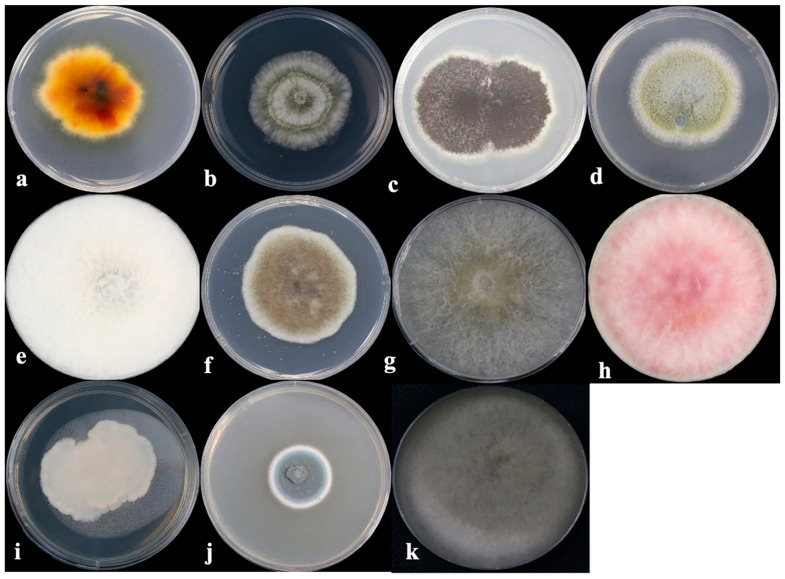
Colonial morphology of 11 isolated fungi grown on PDA at 25 °C for 5 days: (**a**) *Epicoccum* sp., (**b**) *Trichocladium* sp., (**c**) *Aspergillus* sp., (**d**) *Penicillium* sp., (**e**) *Nigrospora* sp., (**f**) *Alternaria* sp., (**g**) *Botrytis* sp., (**h**) *Fusarium* sp., (**i**) *Bipolaris* sp., (**j**) *Cladosporium* sp., (**k**) *Curvularia* sp.

**Figure 2:**
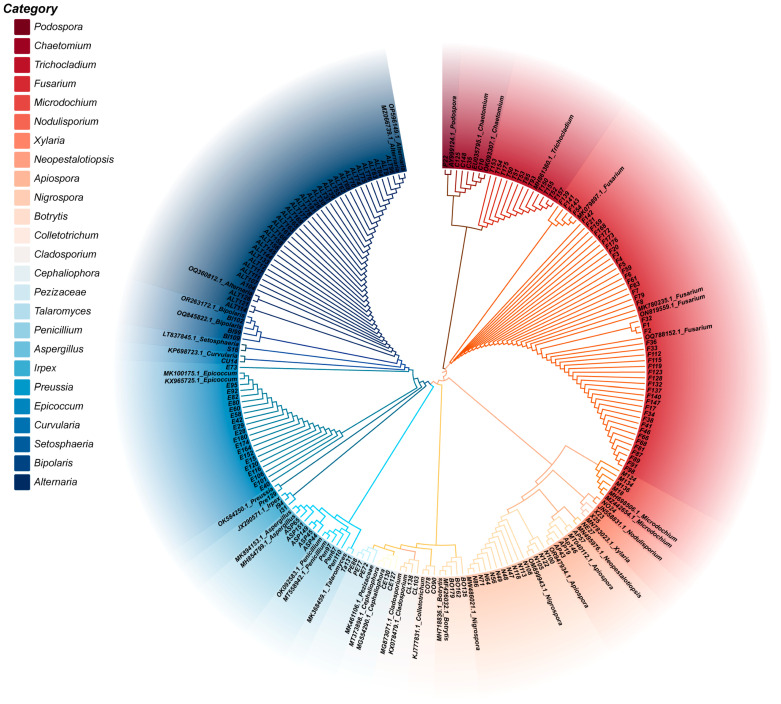
Phylogenetic tree of all the isolated fungi based on the ITS sequence.

A total of 200 fungal strains were isolated as pure fungal cultures from 138 herbal medicine samples collected from different regions in China (Table S1). Through observation of the colony morphology on PDA, combined with PCR amplification of the ITS region and subsequent sequencing, 173 of the obtained fungi were successfully identified and classified into 24 genera (Figure 1). A phylogenetic tree was constructed in the MEGA X software package (10.2, MEGA team, Philadelphia, PA, USA, 2021) [20] by using the neighbor-joining method (Figure 2). According to the ITS sequencing results, Ascomycota (87.43%) was the most abundant phyla, while the predominant genera were *Fusarium* (27.74%) and *Alternaria* (20.81%), followed by *Epicoccum* (11.56%), *Nigrospora* (7.51%), and *Trichocladium* (6.84%) (Table 1).

**Figure 3:**
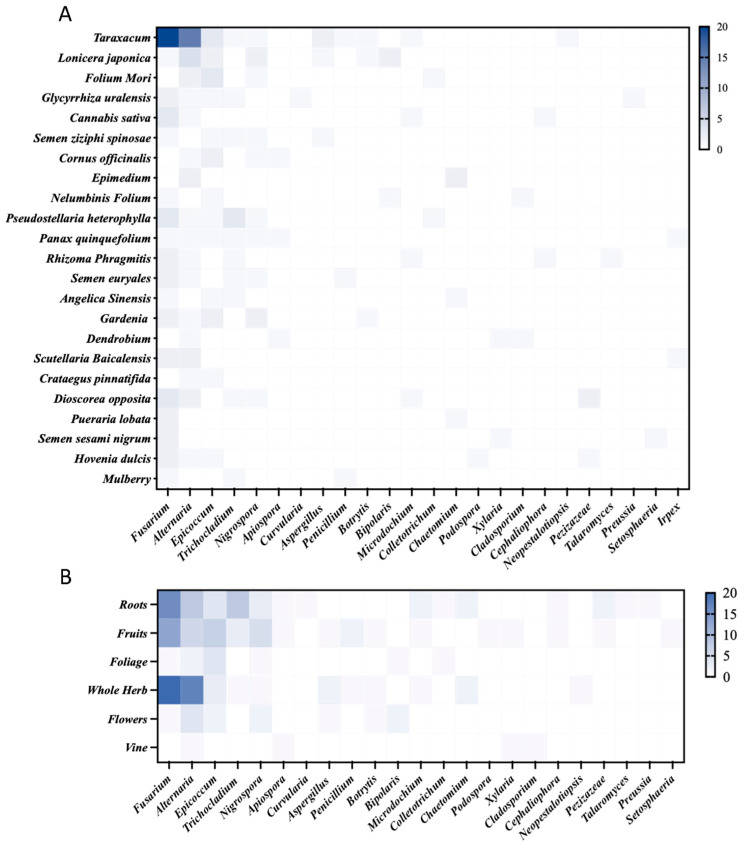
The distribution of fungal species in different kinds (**A**) and types (**B**) of herbal medicines.

The diversity and composition of the fungal communities varied among different herbal medicines. At the phylum level, the overall detection rate of Ascomycota is higher than those of Deuteromycota and Basidiomycota. The abundance of Ascomycota in *Taraxacum mongolicum*, *Pseudostellaria heterophylla*, and *Dioscorea* opposita was significantly higher than that in other medicinal herbs, while the phylum Deuteromycota is more abundant in *Lonicera japonica*, *Gardenia flos*, and *Taraxacum mongolicum*. The only Basidiomycota fungus was isolated from *Scutellaria baicalensis*. *Taraxacum mongolicum* was determined to be the most contaminated herbal medicine, from which a total of 46 fungal strains were obtained; the predominant genera were *Fusarium* (43.48%) and *Alternaria* (32.61%). *Taraxacum mongolicum* is commonly used as a natural antioxidant. Twelve fungal strains were isolated from *Pseudostellaria heterophylla*, and the predominant genus was *Trichocladium* (33.33%), followed by *Fusarium* (25.00%) and *Alternaria* (16.67%). Eleven fungal strains were isolated from *Lonicera japonica*, including *Fusarium* (18.18%), *Alternaria* (18.18%), *Nigrospora* (18.18%), and *Bipolaris* (18.18%) (Figure 3A and Table S2). According to the Pharmacopoeia of the People’s Republic of China, herbal medicines are mainly divided into six types, namely fructus (fruit), radix and rhizome (roots), whole herbs, folium (leaves), blossom (flower), and vine, which are produced from different parts of medicinal herbs. The composition of the fungal communities in different types of herbal medicine was diverse. At the genus level, *Fusarium* was significantly higher in roots, fruits, and whole grasses than other groups, *Alternaria* was the most significant in whole grasses, followed by *Epicoccum* in roots, leaves, and fruits with higher levels than in the other groups. *Trichocladium* was significantly more abundant in the roots, and the abundance of *Nigrospora* was significantly greater in the leaves than in the other groups (Figure 3B). Ascomycota contamination in roots, fruits, and whole herbs was greater than that in other types of herbal medicines, while the distribution of Deuteromycota and Basidiomycota was significantly lower than that of Ascomycota (Figure S1A). The *Taraxacum mongolicum* community was mostly contaminated with Ascomycota (Figure S1B).

### 2.2. Quantitative Analysis of Fusarium and Alternaria Species Found in Herbal Medicines

A qPCR-based detection method was applied in this study for the quantitative analysis of *Fusarium* and *Alternaria* species in herbal medicines randomly collected from different regions of China. The calibration curves for both EF-1α and AQAltpks genes showed good specificity and repeatability (Figure S2), and the LOQs for EF-1α and AQAltpks were 100 and 10 copies/μL, respectively (Figures S3 and S4). The RT-qPCR results showed that all the herbal medicine samples were infected with various amounts of the two abovementioned fungi, with *Taraxacum mongolicum*, *Cannabis sativa*, and *Glycyrrhiza uralensis* being the most severely contaminated herbs (Table 2), within which the *Fusarium* counts reached 32,400–269,000 copies/μL. *Fusarium* contamination in *Lonicera japonica*, *Nelumbinis folium*, *Pseudostellaria heterophylla*, and *Gardenia flos* was also severe, with average counts of 28,966–47,600 copies/μL. The levels of *Fusarium* in other types of medicinal herbs ranged from 2300 to 26,800 copies/μL. The detected content of *Alternaria* was significantly lower than that of *Fusarium* (Table 2), and *Alternaria* contamination was most significant in *Taraxacum mongolicum*, *Pseudostellaria heterophylla*, and *Cornus officinalis*, with average counts of 275–746.78 copies/μL. The contamination levels of *Alternaria* in the other herbs ranged from 25.29 to 303.98 copies/μL.

### 2.3. Mycotoxin Production of the Isolated Fungal Strains

All the *Fusarium* and *Alternaria* strains isolated from herbal medicines in this study were evaluated for their mycotoxin production capacity by cultivation in TBI media. The resulting culture broth was filtered, cleaned, and diluted with 50% acetonitrile solution. A total of 32 mycotoxins were analyzed by LC-MS/MS. Although the LC-MS/MS method we used in this study has not been validated for their usage in herbal medicines, we believe the LC-MS/MS performance is eligible, because we are dealing with the fungal culture in an artificial medium, which is much less complex compared to the natural herbal medicines, so the matrix effect is not as severe as that in the real herbs. As shown in Table 3 and Table 4, seven *Fusarium* sp. and eight *Alternaria* sp. strains among the isolated strains were capable of mycotoxin production. The mycotoxigenic *Fusarium* F1, F59, F63, F54, F172, and F143 were isolated from *Taraxacum mongolicum* and F8 from *Folium mori*. The mycotoxigenic *Alternaria* A177, A181, A158, A26, and A11 were isolated from *Taraxacum mongolicum* and A55, A96, and A62 from *Semen ziziphi spinosae*.

Two strains (F1 and F8) of the mycotoxin-producing *Fusarium* sp. belong to the genus *F. lacertarum*, while three strains (F59, F63, and F172) belong to *F. equiseti*, and the other two (F54 and F143) belong to *F. graminearum*. The *Fusarium* strains produced mainly 3A-DON (40.21~1130.48 μg/kg) and 15A-DON (184.62~4816.12 μg/L), while the content of 15A-DON was greater than that of 3A-DON. F8 is the outlier because it only produced 15A-DON, and it is noteworthy that none of the strains produced DON.

Mycotoxin-producing *Alternaria* sp. belong to four species: strains A177, A181, A55, and A96 were identified as *A. brassicae*; strains A62 and A11 were identified as *A. longipes*; strain A158 was identified as *A. tenuissima*; and strain A26 was identified as *A. arborescens*. ATX-I was the predominant mycotoxin, with concentrations ranging from 665.77 to 7989.39 μg/kg, and all seven strains produced ATX-I. A181, A62, A11, and A26 produced altenuene (ALT); however, it was present at very low concentrations (3.23~9.87 μg/L). Although only A158 and A26 produced alternariol (AOH), the AOH-producing capacity of A26 was relatively high, reaching 406.18 μg/L.

## 3. Discussion

### 3.1. Phytopathogenic Fungi Are Commonly Distributed in Herbal Medicines

Currently, fungal contamination poses a severe threat to the safety of herbal medicine consumption. According to a previous study, fungal contamination of more than 30 commonly used medicinal herbs from southwestern Nigeria was assessed, and high percentages of *Trichocladium*, *Fusarium*, and *Aspergillus* were detected, 34% of which were verified to be able to produce mycotoxins [21]. In addition to phytopathogenic fungal invasion, mycotoxin contamination is vital to the safety of medicinal herbs. In another study, 80% of the samples from 38 medicinal plants available in the Lebanese market were contaminated with 1–11 mycotoxins to varying degrees, with the highest prevalence of FB_1_ (55%), followed by FB_2_ (18%) and OTA (11%) [22]. Chen et al. [23] evaluated the mycoflora and mycotoxin content in 48 herbal medicine samples and revealed that more than 70% of the samples were contaminated with aflatoxins, OTA, and citrinin. In this study, we randomly collected 138 samples of 23 common herbs from different regions of China and identified a total of 173 fungal strains from those herbal samples. *Ascomycota* was the most predominant phylum, and the predominant genera were *Fusarium* (27.43%) and *Alternaria* (20.57%), followed by *Epicoccum* (11.43%), *Nigrospora* (7.43%), and *Trichocladium* (6.86%), indicating that herbs are more susceptible to infestation by ascomycetes such as *Fusarium* and *Alternaria*. Using morphology and ITS sequencing, Guo et al. [24] identified 70 fungal species from 14 *Ziziphi Spinosae Semen* and found that *Aspergillus*, *Candida* and *Wallemia* were the predominant contaminants. Wei et al. [14] revealed that *Aspergillus*, *Fusarium*, and *Cladosporium* were the three predominant genera in 240 herbal medicine samples. Bugno et al. [19] also reported that the presence of *Aspergillus* and *Penicillium* was greater than that of other fungi in Brazilian herbal medicines. However, our results in this study are different from those of other studies to some extent, as we isolated mostly *Fusarium* and *Alternaria* with only *Aspergillus* and *Penicillium*. This phenomenon could be attributed to the culture conditions of the fungi but could also be due to the different hygiene measures used for the samples.

### 3.2. According to RT-qPCR Analysis, Fusarium and Alternaria Contamination Are Severe in Herbal Medicines

Although the molecular identification method surpassed traditional morphological identification, the fungal screening process was still complicated and time-consuming, which hindered us clarifying the mycoflora community in a short period of time. Therefore, we established a real-time qPCR (RT-qPCR) method for the rapid detection of fungal contamination in herbal medicines. RT-qPCR is often used for the detection of fungi in agricultural products. For example, Campos et al. [25] developed two sensitive RT-qPCR TaqMan MGB (Minor Groove Binder) methods for the detection and differentiation of different *Fusarium* species for screening potentially infected maize root systems and confirming that maize was infested with *Fusarium* spp. Roumani et al. [26] established an RT-qPCR assay for the detection of toxin-producing fungi in apples and their byproducts. However, there are fewer examples used for the quantitation of fungi in herbal medicines. According to our isolation and identification of fungi in herbal medicines, we found that *Fusarium* and *Alternaria* were the most serious contaminants; therefore, we chose *Fusarium* and *Alternaria* as the objects of our study, constructed specific primers, and utilized real-time fluorescence to quantitatively detect the occurrence of these two fungi.

### 3.3. Taraxacum mongolicum Is Susceptible to Fungal Contamination

Fungal infestation varies among different types of herbs. Higher levels of fungal infestation can be found in whole herbs, roots, and fruits, and these herb preparation types are more susceptible to fungal colonization because the roots and fruits are rich in starches, oils, and fats, which can provide nutrients for fungal growth [27]. The chemical composition of herbs may also be a major factor influencing fungal communities. The RT-qPCR results also showed that the highest levels of *Fusarium* (27.43%) and *Alternaria* (20.57%) were found in roots, fruits, and whole herbs, which was consistent with the findings of mycorrhizal diversity. In this study, 46 fungal strains, the most strains overall, were isolated from *Taraxacum mongolicum*, and RT-qPCR revealed an abundance of *Fusarium* (269,000 copies/μL) and *Alternaria* (702.37 copies/μL) strains in these herbs. Although other herbs such as *Pseudostellaria heterophylla* and *Glycyrrhiza uralensis* were also severely contaminated with fungi, *Taraxacum mongolicum* was the only herb detected with large amounts of both *Fusarium* and *Alternaria*. Although *Taraxacum mongolicum* is commonly used in China, few studies have investigated its safety, and few studies have focused on its heavy metal residues [28,29,30]. A comprehensive analysis of chemical and biological pollutants in *Taraxacum mongolicum* was performed in Poland, and 14 pathogenic fungi were found by PCR [31]. Our study revealed the potential risk posed by *Taraxacum mongolicum*, but further investigations are needed.

### 3.4. The Isolated Fusarium and Alternaria Strains Are Capable of Mycotoxin Production

*Fusarium* and *Alternaria* are notorious for their mycotoxin-producing abilities, but few studies have focused on the mycotoxin production capacity of the two fungi isolated from herbal medicines. An et al. [32] examined the mycotoxin production ability of the *Fusarium* species from Korean adlay seeds by ELISA analysis, and FUM and ZEN were the main mycotoxin products. However, the isolated *Fusarium* species produced only the acetyl forms of DON in our study, and no other mycotoxins were detected. Zhao et al. [33] evaluated *Alternaria* mycotoxins in herbal medicines by LC-MS/MS and revealed the prevalence of AME and AOH in herbs. In this study, we found that the isolated *Alternaria* strains produced mainly ATX-I, as well as AOH and ALT.

The effects of environmental temperature and humidity on the susceptibility of plants to fungal contamination during planting, harvesting, and storage [34] not only impair the quality of herbal medicine but also pose a potential threat to human health once the risk is not effectively monitored. Herein, we isolated and identified fungi from herbal medicines to clarify the fungal contamination profile and utilized rapid screening for the extent of fungal contamination by RT-qPCR, which provided a basis for the early warning of the risk of fungal contamination in herbal medicines.

## 4. Materials and Methods

### 4.1. Herbal Medicine Sampling

A total of 138 samples of 23 commonly used herbs (6 samples per herb) were collected from 20 regions in China, including radix and rhizome (8 herbs), fructus (9 herbs), folium (2 herbs), blossom (1 herb), whole herb (2 herbs), and vine (1 herb) (Table S1). All samples were split into two halves, one half for fungal isolation and identification, and the other for quantitative detection of *Fusarium* and *Alternaria* in herbal medicines.

### 4.2. Materials and Media Compositions

The authentic samples of 32 kinds of mycotoxins were purchased from Romer Labs GmbH (Getzersdorf, Austria) for the quantitation of mycotoxin contents of the isolated *Fusarium* and *Alternaria*, including deoxynivalenol (DON), 3-acetyl-deoxynivalenol (3ADON), 15-acetyl-deoxynivalenol (15ADON), alterotoxin I (ATX-I), alternuene (ALT), alternariol (AOH), moniliformin (MON), aflatoxins (AFB_1_, AFB_2_, AFG_1_, and AFG_2_), T-2 toxin (T2), hydroxy-T-2 toxin (HT2), diacetoxyscirpenol (DAS), neosolaniol (NEO), beauvericin (BEA), sterigmatocystin (STE), deoxynivalenol 3-glucoside (D3G), DOM [35], enniatins (ENNA, ENNA_1_, ENNB, and ENNB_1_), zearalenone (ZEN), ochratoxin A (OTA), tenuazonic acid (TEA), alternariol 9-monomethyl ether (AME), fumonisins (FB_1_, FB_2_, and FB_3_), nivalenol (NIV), and 4-acetyl-nivalenol (FUX).

LC-MS/MS-grade methanol and acetonitrile were obtained from Merck KGaA (Darmstadt, Germany). All the other chemicals were purchased from a local manufacturer and were of an analytical grade.

Potato Dextrose Agar (PDA): Add 39 g of commercial PDA powder (local manufacturer) to 1 L of distilled water, boil while mixing until dissolved, and autoclave for 20 min at 121 °C. Add 25.0 mg of chloramphenicol to the medium before use to prevent bacterial growth.

Water Agar (WA): Add 1.5 g of agar powder into 1 L of distilled water and autoclave for 20 min at 121 °C.

Trichothecene biosynthesis induction (TBI) medium: Add 30 g sucrose, 1 g KH_2_PO_4_, 0.5 g MgSO_4_ 7H_2_O, 0.5 g KCl, 0.01 g FeSO_4_ 7H_2_O, 1.47 g putrescine hydrochloride, and 100 μL of trace elements solution into 1 L of distilled water, mix until dissolved, and autoclave for 30 min at 115 °C [36].

### 4.3. Isolation of Culturable Fungi from Herbal Medicines

An appropriate amount of herbs was selected and cut into 1–2 cm pieces, which were surface-sterilized in 10% NaClO for 3 min and then rinsed three times with sterile water, then left to dry on the clean bench. The herbs were sparsely inoculated into the PDA disks to be solidified. After the PDA was solidified, the samples were cultured at 25 ℃ for 4–6 days until hyphae growth was observed.

The target hyphae were selected, cut off from the agar disks, then transferred onto WA disks, and culture continued at 25 °C for 24 h–48 h. Then, the single mycelium was selected at the edge of the colony and transferred to PDA disks and cultured at 25 °C for 4–6 days. When the colony grew full mycelium, five pieces of agar blocks were cut out along the edge of the colony. This process was repeated until pure cultures were obtained and stored at 4 °C for further observation and identification. When the subsequent operations were completed, six pieces of agar were cut out along the edge of the colony, stored in 2 mL cryogenic vials containing PDA or 30% glycerol, and finally stored at −80 °C.

### 4.4. Molecular Identification of the Isolated Fungi

Fresh mycelium was placed into a 1.5 mL centrifuge tube, then 300 μL of PBS (pH 7.0) was added, and the glass rod was used to grind the mycelium along the wall of the centrifugal tube to obtain the turbid mycelium homogenate. The purified genomic DNA of the fungal strains was then extracted using the UE Multisource Genomic DNA Minprep Kit (UE Landy Biotechnology Co., Ltd., Suzhou, China), and the obtained DNA were stored at −80 ℃.

The primers for the ITS of the fungi were ITS1(5′-TCCGTAGGTGAACCACCTGCGC-3′) and ITS4(5′-TCCTCCGCTTA TTGATATGC-3′).

Polymerase chain reaction (PCR) was performed in a 25 μL reaction mixture solution containing 2 μL of DNA, 0.5 μL each of the forward and reverse primers, 9.5 μL of double-distilled water, and 12.5 μL of 2× Power Taq PCR Master Mix (Takara Biotechnology Co., Ltd.). The amplification program comprised initial denaturation at 95 °C for 5 min, followed by 35 cycles of denaturing at 94 °C for 40 s, annealing at 53 °C for 40 s, extension at 72 °C for 1 min by 35 cycles, and a final extension for 10 min at 72 °C. PCR amplification products were verified by 1.5% agarose gel electrophoresis. The amplified fragments were purified and sequenced by an external service provider (Sangon Biotech Co., Ltd., Shanghai, China). The sequences were used to perform a BLAST search against the National Center for Biotechnology Information (NCBI) sequence database (GenBank: https://www.ncbi.nlm.nih.gov (accessed on 14 May 2024)) to compare the sequences with the most similarity to identify each fungus. Finally, a phylogenetic tree was constructed with the neighbor-joining method using the MEGA X software package (10.2, MEGA team, Philadelphia, PA, USA, 2021) [20].

### 4.5. Extraction of Fungal Genomic DNA from Herbal Medicines

Fungal genomic DNA from the crude herbal medicine samples were extracted by using the cetyltrimethylammonium bromide (CTAB) method. The whole process was applied following the instruction of the CTAB Plant Genome DNA Kit (Beijing Biomed Gene technology Co., Ltd., Beijing, China). The obtained genomic DNA were stored at −80 °C.4.6. Real-Time PCR Method for Detecting *Fusarium* and *Alternaria* in Herbal Medicines

Primers for quantitative detection of *Fusarium* species were designed according to the conserved sequence of the translation elongation factor 1α (*EF-1α*) gene of *Fusarium*, which were AQFF1 (5′-atgaccatgattacgccaATAGGAAGCCGCTGAGCTCGGTAA-3′) and AQFR1 (5′-gtaaaacgacggccagtgACCAATGACGGTGACATAGTAGCG-3′) [37]. Specific primers were synthesized for the quantitative detection of *Alternaria* species utilizing the polyketide synthase gene (*PksH*) which is required for toxin synthesis [38]. The primers were *AQAltpksHF* (5′-atgaccatgattacgccaGTCAACCCTCTCACACCAAC-3′) and *AQAltpksHR* (5′-gtaaaacgacggccagtgGACGCATCGCTTCAATAGCC-3′).

To construct the standard plasmids for calibration, conventional PCR amplification of *Fusarium* or *Alternaria* DNA obtained from the herbs was performed using these primers as previously described. DNA products were then subjected to 1% agarose gel electrophoresis, recovered, and purified using a SanPrep Column Plasma Mini Preps Kit (Sangon Biotech Co., Ltd., Shanghai, China). The fragments were purified and cloned into the vector pUC19 using the Uniclone One Step Seamless Cloning Kit (Genesand, Beijing, China) via homologous recombination at the restriction sites *HindШ* and *EcoRI*. The recombinant plasmid was transformed into an Efficom5α Chemically Competent Cell. The transformed Efficom5α cells were coated in solid LB medium overnight, and the resulting single colonies were inoculated into liquid LB medium and shaken at 220 rpm for 12 h at 37 °C. Following clone PCR verification, the positive clones were sequenced by an external service provider (Sangon Biotech Co., Ltd., Shanghai, China), and the obtained sequences were compared with the NCBI database. Plasmids were extracted using the SanPrep Column Plasmid Mini-Preps Kit (Sangon Biotech Co., Ltd., Shanghai, China) and quantified using NanoVue Plus™ (GE, Newark, NJ, USA). The standard curve was constructed using serial ten-fold dilutions of the positive recombinant plasmid. The real-time quantitative PCR reaction system (20 μL) was composed of 0.5 μL each of the forward and reverse primers, 2 μL of template DNA, and 12.5 μL of TB Green *premix Ex Taq* II. Furthermore, PCR was performed under initial denaturation at 95 °C for 2 min, 45 cycles of melting at 95 °C for 10 s, and annealing at 56 °C for 20 s. As a negative control, ddH_2_O was used in place of the template. Fluorescence signals were monitored during the annealing phase of each PCR cycle, and Ct values were obtained. The concentration of *Fusarium* or *Alternaria* DNA in the herbal medicines was determined on a Roche LightCycler 96 system (Basel, Switzerland).

### 4.6. Mycotoxin Production Assay

All the fungal isolates belonging to the *Fusarium* and *Alternaria* species were compared for their mycotoxin production. Each fungus preserved in the freezer was inoculated on PDA and cultured at 25 °C for 5 d, then the mycelium was picked up by a toothpick and inoculated into 30 mL of mungbean medium for sporation. After incubating at 25 °C and 180 rpm under lighting for 5 days, the spores were collected by centrifugation. Conidia (10^5^) of each *Fusarium* or *Alternaria* fungus were inoculated into 30 mL of TBI medium and cultured in the dark at 25 °C and 180 rpm for 7 d. The supernatant of the medium was filtered, lyophilized, and dissolved in 50% acetonitrile solution for LC-MS/MS analysis. The chromatographic parameters were in accordance with our previous report [39]. Briefly, an AB Sciex 3500 LC–MS/MS system was used to simultaneously quantify mycotoxins. The XDB-C18 analytical column (2.1 × 150 mm, 3.5 μm bead diameter; Agilent) was used, and the column temperature was held at 30 °C. A gradient elution was applied as follows: 0.5 mL/min 10% B, 2 min 50% B, 4 min 60% B, 5 min 95% B, 8 min 95% B, and 8.1 min 10% B (A, water with 0.1% acetic acid, B, methanol). Nitrogen was used as the drying gas (10 L/min). The capillary voltage was set at 4 kV, the nebulizer pressure 30 psi, and the drying gas temperature 300 °C. Mycotoxins were analyzed via multiple reaction monitoring. A total of 32 kinds of mycotoxins were quantified by multiple reaction mode (MRM). 

## Figures and Tables

**Table 1:** Frequency of contaminated fungi isolated from herbal medicines.

Genera	Counts	Frequency (%)
*Fusarium*	48	27.75
*Alternaria*	36	20.81
*Epicoccum*	20	11.56
*Nigrospora*	13	7.51
*Trichocladium*	12	6.94
*Aspergillus*	5	2.89
*Chaetomium*	4	2.31
*Microdochium*	4	2.31
*Apiospora*	3	1.73
*Botrytis*	3	1.73
*Cladosporium*	3	1.73
*Pezizaceae*	3	1.73
*Penicilium*	3	1.73
*Bipolaris*	3	1.73
*Xylaria*	2	1.16
*Colletotrichum*	2	1.16
*Irpex*	2	1.16
*Neopestalotiopsis*	1	0.58
*Nodulisporium*	1	0.58
*Podospora*	1	0.58
*Talaromyces*	1	0.58
*Preussia*	1	0.58
*Curvularia*	1	0.58
*Setosphaeria*	1	0.58
Total	173	100

**Table 2:** Detection of *Fusarium* sp. and *Alternaria* sp. from herbal medicine samples. The quantitation was achieved by using qRT-PCR with species-specific primers for *Fusarium* sp. and *Alternaria* sp., respectively.

Samples	Source	*Fusarium* Counts (Copies/μL)	*Alternaria* Counts (Copies/μL)
*Semen euryales*	ZhaoQing, Guangdong	4433.33 ± 1412.13	70.66 ± 1.96
	Jining, Shandong	7366.67 ± 1136	28.62 ± 4.75
*Scutellaria baicalensis*	Yuncheng, Shanxi	11,800.00 ± 1808	35.22 ± 9.30
	Taizhou, Jiangsu	19,343.30 ± 935.80	26.08 ± 4.81
*Mulberry* *fructus*	Yancheng, Jiangsu	7123.33 ± 1346.21	35.05 ± 2.95
	Guangyuan, Sichuan	14,400.03 ± 916.5	56.77 ± 12.35
*Taraxacum mongolicum*	Taizhou, Jiangsu	122,667.06 ± 18,877.14	678.07 ± 53.09
	Tianshui, Gansu	269,020.40 ± 10,003.12	327.67 ± 7.92
*Lonicera japonica*	Linyi, Shandong	41,203.00 ± 2666.62	33.69 ± 5.69
	Suiyang, Guizhou	41,711.30 ± 2883.51	44.76 ± 8.39
*Angelica sinensis*	Longnan, Gansu	13,407.05 ± 2007.40	33.31 ± 7.71
	Yaan, Sichuan	7750.00 ± 1192.31	40.17 ± 6.25
*Semen ziziphi spinosae*	Xingtai, Hebei	4773.33 ± 1590.23	668.51 ± 62.00
	Taizhou, Jiangsu	5876.67 ± 1262.17	289.81 ± 55.11
*Panax quinquefolium*	Xinbin, Liaoning	5656.67 ± 491.70	26.56 5.08
	Fusong, Jilin	14,033.30 ± 814.50	33.23 ± 6.09
*Crataegus pinnatifida*	Yinan, Shandong	3025.16 ± 1160.25	177.58 ± 5.22
	Nanyang, Henan	3071.67 ± 855.41	303.98 ± 17.25
*Epimedium brevicornum* Maxim.	Taizhou, Jiangsu	33,133.30 ± 3147.20	33.03 ± 3.35
	Xihe, Gansu	24,100.00 ± 3005.28	25.30 ± 3.06
*Cannabis sativa*	Taizhou, Jiangsu	24,921.25 ± 4089.30	24.91 ± 4.11
	Jincheng, Shanxi	159,667.12 ± 9292.21	38.92 ± 3.11
*Folium mori*	Taizhou, Jiangsu	32,410.21 ± 5671.13	34.23 ± 3.75
	Yancheng, Jiangsu	22,933.30 ± 2811.15	48.14 ± 3.03
*Nelumbinis folium*	Fuzhou, Jiangxi	42,633.42 ± 3617.05	56.00 ± 6.00
	Caoxian, Shandong	40,966.70 ± 3086.75	50.60 ± 7.308
*Gardenia flos*	Fuding, Fujian	41,632.24 ± 7569.23	204.84 ± 30.29
	Jiujiang, Jiangxi	38,952.18 ± 919.20	173.00 ± 7.55
*Pseudostellaria heterophylla*	Taizhou, Jiangsu	47,601.13 ± 1414.06	270.96 ± 15.14
	Zherong, Fujian	28,966.70 ± 5338.21	164.6 ± 13.01
*Cornus officinalis*	Luoyang, Henan	8467.33 ± 553.70	746.78 ± 44.66
	Taizhou, Jiangsu	16,566.70 ± 5179.40	275.00 ± 29.21
*Glycyrrhiza uralensis*	Taizhou, Jiangsu	112,524.61 ± 12,021.28	161.61 ± 13.58
	Longxi, Gansu	43,512.93 ± 5620.63	288.33 ± 19.14
*Dioscorea opposita*	Yulin, Guangxi	26,366.71 ± 6962.43	38.80 ± 3.71
	Jiaozuo, Henan	27,126.19 ± 3412.35	65.23 ± 7.38
*Rhizoma phragmitis*	Xuzhou, Jiangsu	5045.67 ± 614.60	27.98 ± 1.71
	Shangqiu, Henan	2783.33 ± 328.11	58.44 ± 11.47
*Dendrobium officinale*	Huoshan, Anhui	7330.51 ± 1245.27	164.97 ± 16.34
	Dehong, Yunnan	6163.32 ± 242.10	188.46 ± 37.40
*Hovenia dulcis*	Dushan, Guizhou	19,902.13 ± 3351.25	26.58 ± 1.98
	Ankang, Shanxi	26,802.61 ± 4314.76	51.71 ± 4.91
*Semen sesami nigrum*	Fuyang, Anhui	2301.24 ± 495.40	30.37 ± 5.53
	Zhoukou, Henan	7005.71 ± 1011.65	34.50 ± 5.98
*Pueraria lobata*	Tengxian, Guangxi	3383.41 ± 416.20	27.79 ± 1.86
	Taizhou, Jiangsu	5746.67 ± 320.8	68.50 ± 4.65

**Table 3:** Mycotoxigenic capacity of the isolated *Fusarium* strains (mycotoxin detected in μg/kg).

Species	F1	F8	F59	F63	F172	F54	F143
	*F. lacertarum*	*F. lacertarum*	*F. equiseti*	*F. equiseti*	*F. equiseti*	*F. graminearum*	*F. graminearum*
3ADON	40.21 ± 5.35	-	253.09 ± 6.52	127.56 ± 4.49	138.51 ± 6.52	1130.48 ± 53.45	703.28 ± 62.85
15ADON	245.28 ± 8.41	184.62 ± 12.67	1156.74 ± 35.83	483.61 ± 14.29	394.92 ± 17.26	4816.12 ± 105.71	2874.73 ± 52.53

The contents of DON, ATX-I, ALT, AOH, MON, AFB1, AFG1, AFB2, AFG2, T2, HT2, DAS, NEO, BEA, STE, D3G, DOM, ENNA, ENNA1, ENNB, ENNB1, ZEN, OTA, TEA, AME, FB1, FB2, FB3, NIV, and FUX were all below the LOD according to LC-MS/MS analysis.

**Table 4:** Mycotoxigenic capacity of the isolated *Alternaria* strains (mycotoxin detected in μg/kg).

Species	A177	A181	A55	A96	A158	A62	A11	A26
	*A. brassicae*	*A. brassicae*	*A. brassicae*	*A. brassicae*	*A. tenuissima*	*A. longipes*	*A. longipes*	*A. arborescens*
ATX-I	2431.15 ± 188.26	2162.61 ± 108.99	3264.83 ± 129.15	3215.79 ± 49.45	7989.39 ± 88.05	665.77 ± 33.85	840.37 ± 14.84	1218.49 ± 34.39
ALT	-	8.26 ± 0.37	-	-	-	4.47 ± 0.42	3.23 ± 0.85	9.87 ± 1.54
AOH	-	-	-	-	38.98 ± 1.18	-	-	406.18 ± 13.71

The contents of DON, 3ADON, 15ADON, MON, AFB1, AFG1, AFB2, AFG2, T2, HT2, DAS, NEO, BEA, STE, D3G, DOM, ENNA, ENNA1, ENNB, ENNB1, ZEN, OTA, TEA, AME, FB1, FB2, FB3, NIV, and FUX were all below the LOD according to LC-MS/MS analysis.

## Data Availability

The data will be made available upon request.

## Supplementary Materials

The following supporting information can be downloaded at https://www.mdpi.com/article/10.3390/toxins16050229/s1, Figure S1: Contamination assessment of phytopathogenic fungi on different kinds of herbal medicines on a phylum level; Figure S2: Sensitivity of the RT-qPCR assay for detection of *Fusarium* spp. and *Alternaria* spp.; Figure S3: The calibration curve and dissociation curve of recombinant plasmid amplification products for *Fusarium EF-1α* gene; Figure S4: The external calibration curve and dissociation curve of recombinant plasmid amplification products for *Alternaria AQAltpks* gene; Figure S5: Colonial morphology of all the 24 genera of isolated fungi grown on PDA at 25 °C for 5 days; Table S1: Sources of the herbal medicines used in this study; Table S2: Frequency of fungal contamination from different herbal medicines; Table S3: Frequency of fungal contamination from *Taraxacum mongolicum*.
