# Supplementary material for: Quantitative Analysis of Fungal Contamination of Different Herbal Medicines in China
Source: Toxins (Basel). 2024 May 15;16(5):229. doi: 10.3390/toxins16050229 (PMC11126118; doi:10.3390/toxins16050229)
Supplement: Supplementary file 1 [file toxins-16-00229-s001.zip › toxins-2991464-supplementary.pdf]

Supplementary Materials: Quantitative Analysis of Fungal Contamination of Different Herbal Medicines in China

Gang Wang [1,2], Mingyue Jiao [1,2], Junqiang Hu [2,3], Yiren Xun [1,2], Longyun Chen [1,2], Jianbo Qiu [2], Fang Ji [2], Yin-Won Lee [4], Jianrong Shi [2] and Jianhong Xu [1,2,*]

[1] School of Food and Biological Engineering, Jiangsu University, Zhenjiang 212013, China; wanggang2015@jaas.ac.cn (G.W.); jiaomy2021@163.com (M.J.); xunyr2017@163.com (Y.X.); clyun99@163.com (L.C.)

[2] Jiangsu Key Laboratory for Food Quality and Safety-State Key Laboratory Cultivation Base, Ministry of Science and Technology/Key Laboratory for Agro-product Safety Risk Evaluation (Nanjing), Ministry of Agriculture and Rural Affairs/Key Laboratory for Control Technology and Standard for Agro-Product Safety and Quality, Ministry of Agriculture and Rural Affairs/Collaborative Innovation Center for Modern Grain Circulation and Safety/Institute of Food Safety and Nutrition, Jiangsu Academy of Agricultural Sciences, Nanjing 210014, China; 2021216027@stu.njau.edu.cn (J.H.); 20120027@jaas.ac.cn (J.Q.); jifang625@126.com (F.J.); jianrong63@126.com (J.S.)

[3] Key Laboratory of Agricultural Environmental Microbiology, Ministry of Agriculture, College of Life Sciences, Nanjing Agricultural University, Nanjing 210095, China

[4] Department of Agricultural Biotechnology, Seoul National University, Seoul 08826, Republic of Korea; lee2443@snu.ac.kr

* Correspondence: xjh@jaas.ac.cn

Table S1. Sources of the herbal medicines used in this study

Category	Samples (n)	Place of origin
Fructus	*Crataegus pinnatifida* (6)	Yinan, Shandong / Nanyang, Henan
	Gardenia flos (6)	Fuding, Fujian / Jiujiang, Jiangxi
	Semen euryales (6)	Zhaoqing, Guangdong / Jining, Shandong
	Semen sesami nigrum (6)	Fuyang, Anhui / Zhoukou, Henan
	Hovenia dulcis (6)	Dushan, Guizhou / Ankang, Shanxi
	Mulberry (6)	Yancheng, Jiangsu / Guangyuan, Sichuan
	Cannabis sativa (6)	Taizhou, Jiangsu / Jincheng, Shanxi
	Semen ziziphi spinosae (6)	Taizhou, Jiangsu / Xingtai, Hebei
	Cornus officinalis (6)	Taizhou, Jiangsu / Luoyang, Henan
Radix and rhizome	*Pseudostellaria heterophylla* (6)	Taizhou, Jiangsu / Zherong, Fujian
	Panax quinquefolium (6)	Xinbin, Liaoning / Fusong, Jilin
	Rhizoma phragmitis (6)	Xuzhou, Jiangsu / Shangqiu, Henan
	Glycyrrhiza uralensis (6)	Taizhou, Jiangsu / Longxi, Gansu
	Angelica sinensis (6)	Longnan, Gansu / Yaan, Sichuan
	Scutellaria baicalensis (6)	Taizhou, Jiangsu / Yuncheng, Shanxi
	Dioscorea opposita (6)	Jiaozuo, Henan / Yulin, Guangxi
	Pueraria lobata (6)	Taizhou, Jiangsu / Tengxian, Guangxi
Whole herbs	*Taraxacum mongolicum* (6)	Taizhou, Jiangsu / Tianshui, Gansu
	Epimedium brevicornum Maxim. (6)	Taizhou, Jiangsu / Xihe, Gansu
Folium	*Folium mori* (6)	Yancheng, Jiangsu / Taizhou, Jiangsu
	Nelumbinis folium (6)	Fuzhou, Jiangxi / Caoxian, Shandong
Blossom	*Lonicera japonica* (6)	Linyi, Shandong / Suiyang, Guizhou
Vine	*Dendrobium officinale* (6)	Dehong, Yunnan / Huoshan, Anhui

Table S2. Frequency of fungal contamination from different herbal medicines

Herbs	Counts	Frequency (%)
Taraxacum mongolicum	46	26.59
Lonicera japonica	11	6.36
Folium mori	7	4.05
Glycyrrhiza uralensis	7	4.05
Cannabis sativa	6	3.47
Semen ziziphi spinosae	5	2.89
Cornus officinalis	5	2.89
Epimedium brevicornum Maxim.	4	2.31
Nelumbinis folium	4	2.31
Pseudostellaria radix	10	5.78
Panax quinquefolium radix	7	4.05
Rhizoma phragmitis	7	4.05
Semen euryales	6	3.47
Angelica sinensis radix	4	2.31
Gardenia flos	8	4.62
Dendrobium officinale	4	2.31
Scutellaria baicalensis	3	1.73
Crataegus pinnatifida	2	1.16
Dioscorea opposita	10	5.78
Pueraria lobata	2	1.16
Semen sesami nigrum	4	2.31
Hovenia dulcis	7	4.05
Mulberry fructus	4	2.31
Total	173	100

Table S3. Frequency of fungal contamination from *Taraxacum mongolicum*

Category	Place of origin							
	Taizhou, Jiangsu	Tianshui, Gansu	Yuncheng, Shanxi	Zhengzhou, Henan	Linyi, Shandong	Hangzhou, Zhejiang	Yulin, Guangxi	Luan, Anhui
Fusarium spp.	5	3	2	2	3	2	2	1
Alternaria spp.	2	4	1	-	2	-	3	3
Epicoccum spp.	-	1	1	-	-	1	-	-
Nigrospora spp.	1	-	-	-	-	-	-	-
Trichocladium spp.		-	1	-	-	-	-	-
Aspergillus spp.	1	-	-	1	-	-	-	-
Microdochium spp.	-	-	1	-	-	-	-	-
Botrytis spp.	-	-	-	-	1	-		
Penicilium spp.	1	-	-	-	-	-	-	-
Neopestalotiopsis spp.	-	-	-	-	-	1	-	-

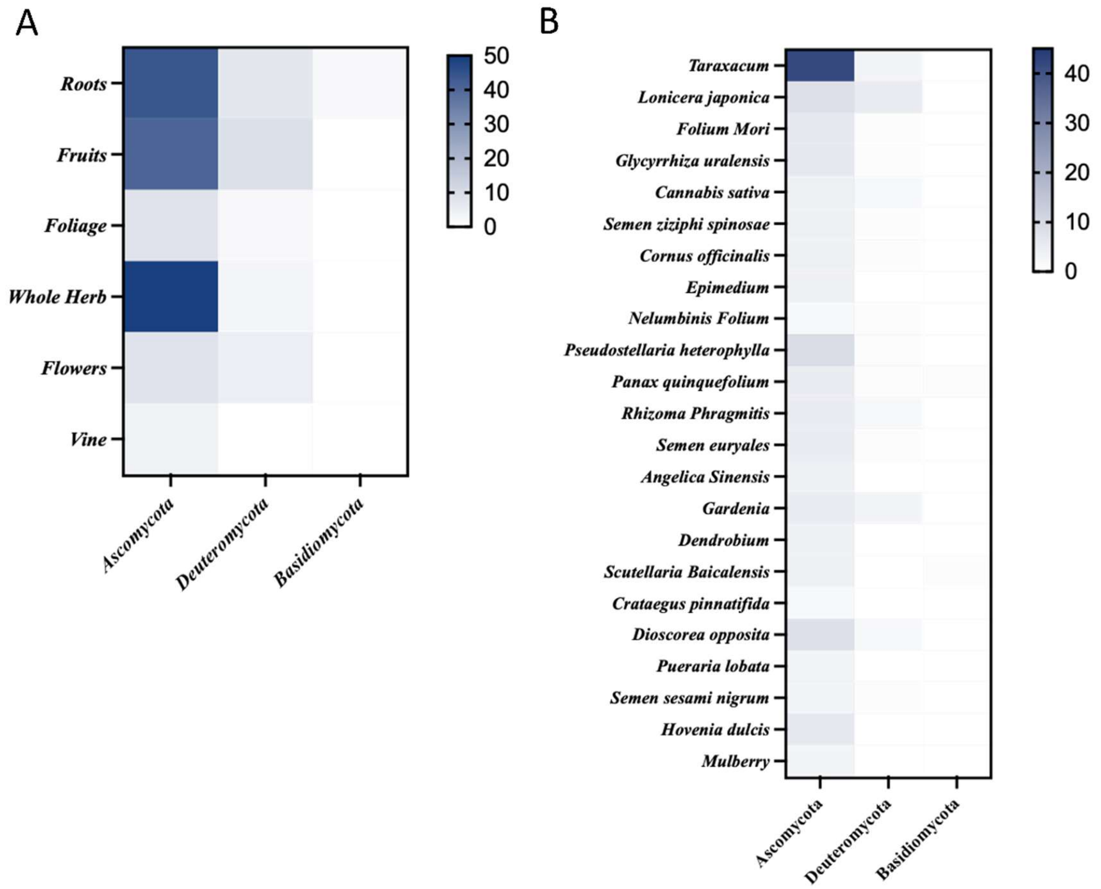

Figure S1. Contamination assessment of phytopathogenic fungi on different kinds of herbal medicines on a phylum level.

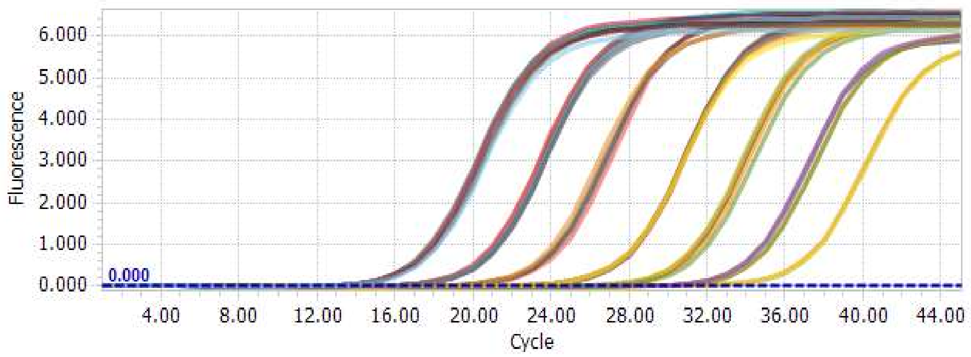

(A)

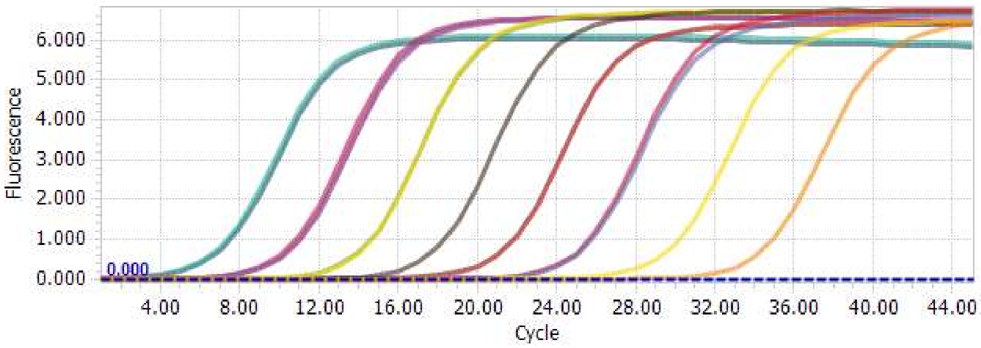

(B)

Figure S2. Sensitivity of the RT-qPCR assay for detection of *Fusarium* spp. (A) and *Alternaria* spp. (B)

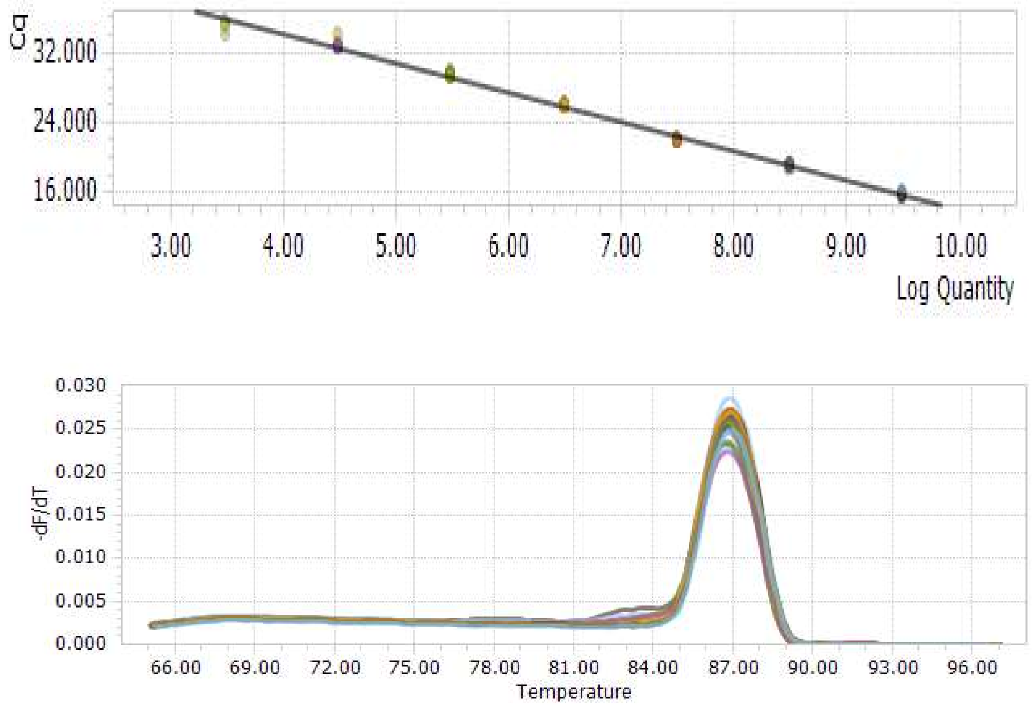

Figure S3. The calibration curve and dissociation curve of recombinant plasmids amplification products for *Fusarium EF-1α* gene

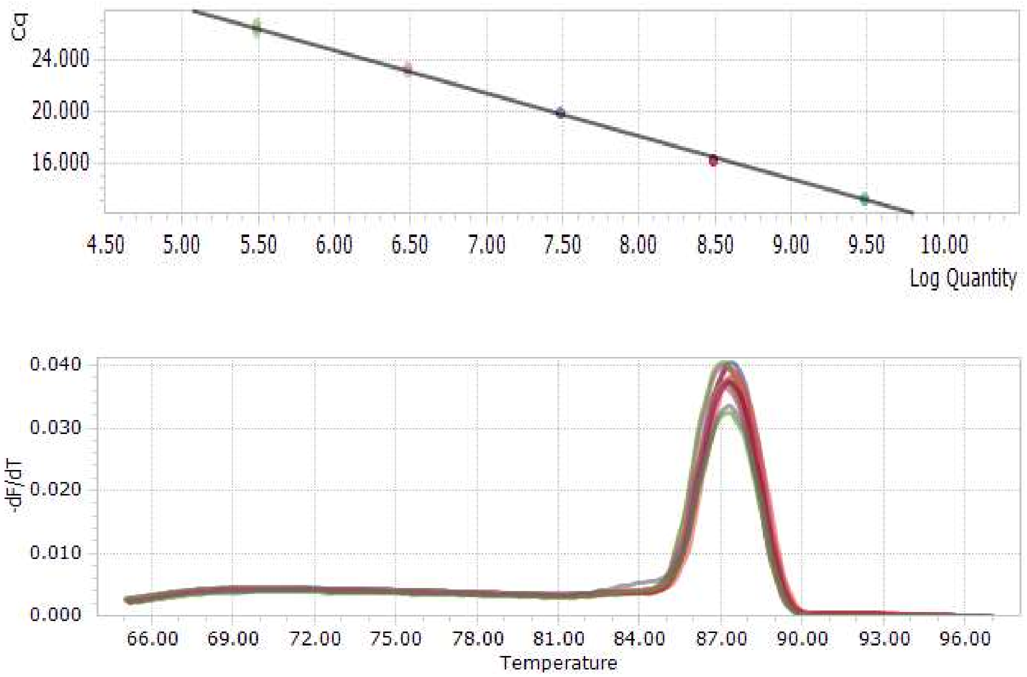

Figure S4. The external calibration curve and dissociation curve of recombinant plasmids amplification products for *Alternaria AQAltpks* gene

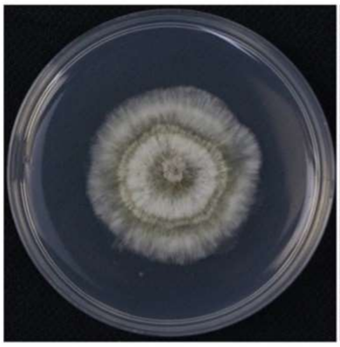

Trichocladium sp.

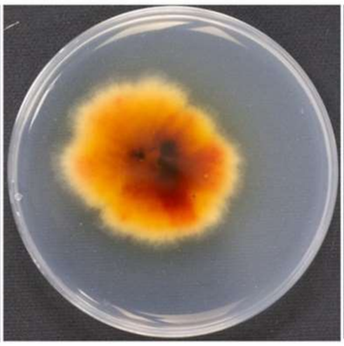

Epicoccum sp.

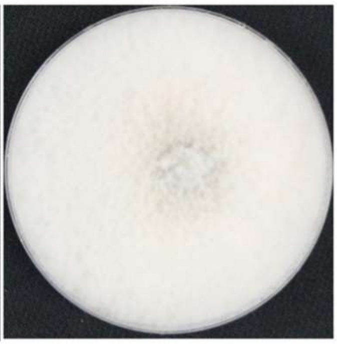

Nigrospora sp.

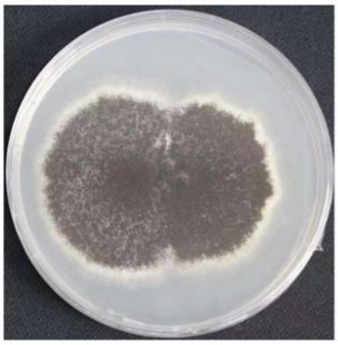

Aspergillus sp.

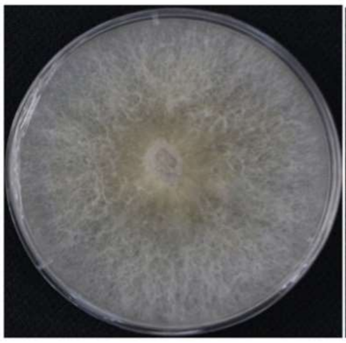

Botrytis sp.

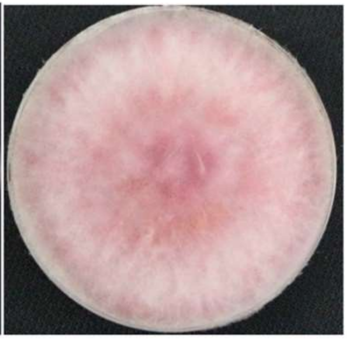

Fusarium sp.

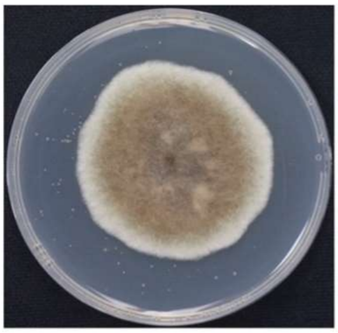

Alternaria sp.

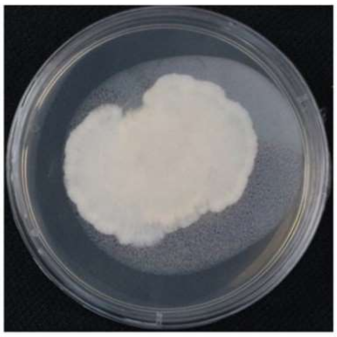

Bipolaris sp.

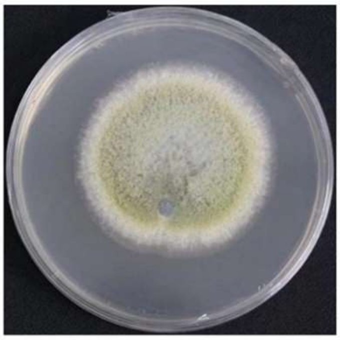

Penicilium sp.

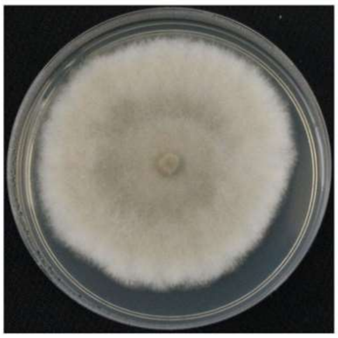

Curvularia sp.

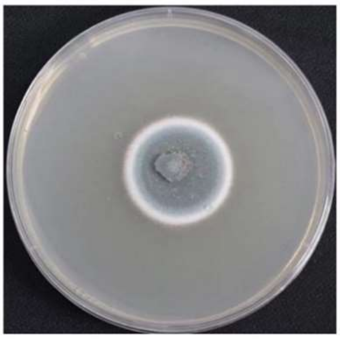

Cladosporium sp.

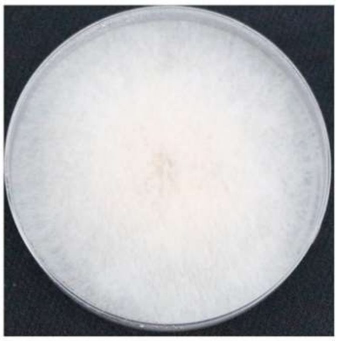

Apiospora sp.

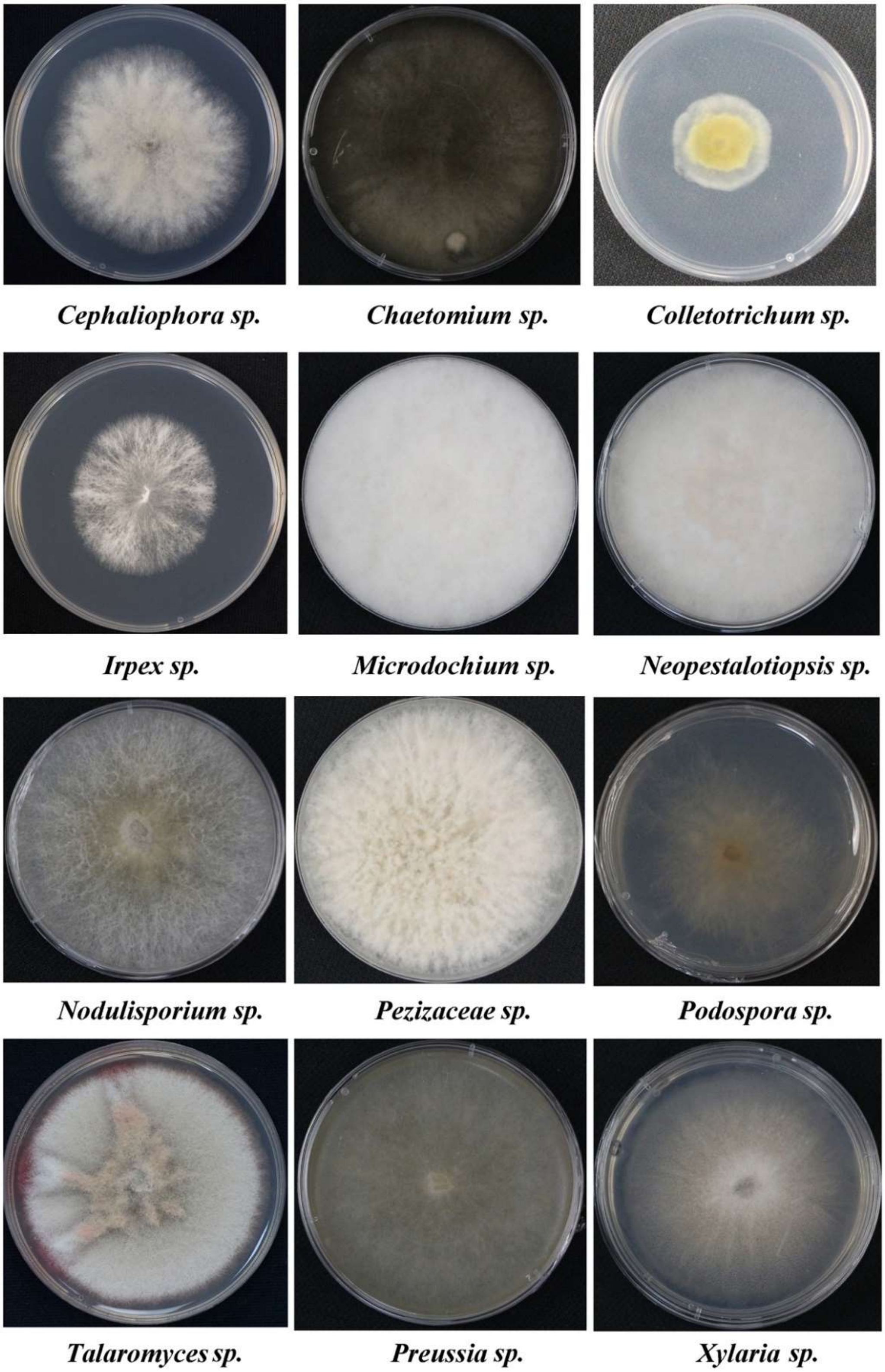

Figure S5. Colonial morphology of all the 24 genera of isolated fungi grown on PDA at 25 °C for 5 days
